# Cabozantinib: an Active Novel Multikinase Inhibitor in Renal Cell Carcinoma

**DOI:** 10.1007/s11912-017-0566-9

**Published:** 2017-02-28

**Authors:** Nizar M. Tannir, Gisela Schwab, Viktor Grünwald

**Affiliations:** 1 Department of Genitourinary Medical Oncology, The University of Texas MD Anderson Cancer Center, 1155 Pressler St., Unit 1374, Houston, TX 77030 USA; 2 Exelixis, Inc., 210 E. Grand Avenue, South San Francisco, CA 94080 USA; 3 Departments of Hematology, Hemostasis, Oncology, and Stem Cell Transplantation, Medical School Hannover (MHH), OE6860 Carl-Neuberg-Str. 1, 30625 Hannover, Germany

**Keywords:** Renal cell carcinoma, Cabozantinib, Angiogenesis, VEGF receptor, MET, AXL, RCC

## Abstract

Clear cell renal cell carcinoma (RCC) is characterized by inactivation of the von Hippel-Lindau (VHL) tumor suppressor gene. VHL loss drives tumor angiogenesis and accounts for the clinical activity of VEGF receptor (VEGFR) tyrosine kinase inhibitors (TKIs), the first-line standard of care for advanced RCC. Within the last year, three new second-line treatments have received FDA approval for use after anti-angiogenic therapy: the immune checkpoint inhibitor nivolumab, the TKI cabozantinib, and the combination of the TKI lenvatinib and the mTOR inhibitor everolimus. Cabozantinib inhibits VEGFRs, MET, and AXL, kinases that promote tumorigenesis, angiogenesis, metastasis, and drug resistance. Compared with everolimus, cabozantinib has shown statistically significant improvements in the three key efficacy endpoints of overall survival, progression-free survival, and objective response rate in patients with RCC who were previously treated with a VEGFR TKI. Herein, we summarize the translational research and clinical development that led to approval of cabozantinib as second-line therapy in RCC.

## Introduction

Renal cell carcinoma (RCC), the most common form of kidney cancer, comprises several malignancies that arise from renal tubular epithelial cells; clear cell RCC is the most common histological subtype accounting for 80–90% of cases [1]. The yearly incidence of RCC is roughly 300,000 worldwide and more than 50,000 in the USA [2, 3]. The disease occurs twice as frequently in men as in women. Approximately 70% of RCC cases are diagnosed in patients ≥50 years of age, with a median age of 64 years at diagnosis [2, 4]. Risk factors for the development of RCC include smoking and obesity [5–7]. RCC cases are almost always sporadic ( ~96%) but can be familial ( ~4%) and are often linked to specific gene mutations [8].

When diagnosed at an early stage (>50% of cases), patients with RCC have a favorable prognosis following nephrectomy with a 5-year survival rate of 81% for stage I disease [9–11]. However, 10–30% of patients with early-stage disease will experience tumor recurrence following resection [12–14], and 20–30% of patients present with stage IV metastatic disease [15]. For advanced disease, systemic therapy is the foundation of treatment, but long-term prognosis is poor with a 5-year survival rate of approximately 12% during the period 2004–2010 [10, 11].

The most common genetic lesion in both the familial and sporadic forms of clear cell RCC is inactivation of the von Hippel-Lindau (VHL) tumor suppressor gene [16]. VHL disruption results in highly angiogenic and vascularized tumors [17]. Thus, development of systemic treatments has focused on the angiogenic axis. A number of targeted therapies have been approved for use in RCC, including tyrosine kinase inhibitors (TKIs) with anti-angiogenic activity (sunitinib, pazopanib, sorafenib, and axitinib), the VEGF-targeted monoclonal antibody bevacizumab, and the mTOR inhibitors everolimus and temsirolimus [11]. In the past, immunotherapy with high-dose interleukin-2 played an important role in the treatment of patients with metastatic clear cell RCC who have excellent performance status and normal organ function, but due to its inherent toxicity and the availability of alternative systemic therapies, its use now is declining [11].

In the past year, three new second-line treatments for RCC have been approved by the FDA—the immune checkpoint inhibitor nivolumab [18••], the TKI lenvatinib in combination with the mTOR inhibitor everolimus [19••, 20], and the TKI cabozantinib, which is the focus of this review [21••, 22••].

Cabozantinib is an orally bioavailable TKI which targets VEGF receptors (VEGFRs), MET, AXL, and other receptor tyrosine kinases involved in tumor development and progression through angiogenesis, anti-apoptosis, invasiveness, metastasis, and drug resistance [23]. Cabozantinib has been evaluated in a number of solid tumors including medullary thyroid cancer, castration-resistant prostate cancer, and non-small cell lung cancer [24–28]. In a randomized phase 3 trial for progressive metastatic medullary thyroid cancer, cabozantinib treatment significantly improved progression-free survival (PFS) and objective response rate (ORR) compared with placebo leading to regulatory approval in this indication [28]. Recently, cabozantinib demonstrated improvements in PFS, ORR, and overall survival compared with everolimus in patients with advanced RCC who had received prior anti-angiogenic therapy [21••, 22••]. Herein, we provide an overview of the clinical development of cabozantinib in RCC, from translational research through the pivotal phase 3 trial that supported its regulatory approval.

## Disease Mechanism and Drug Resistance in RCC

**Fig. 1:**
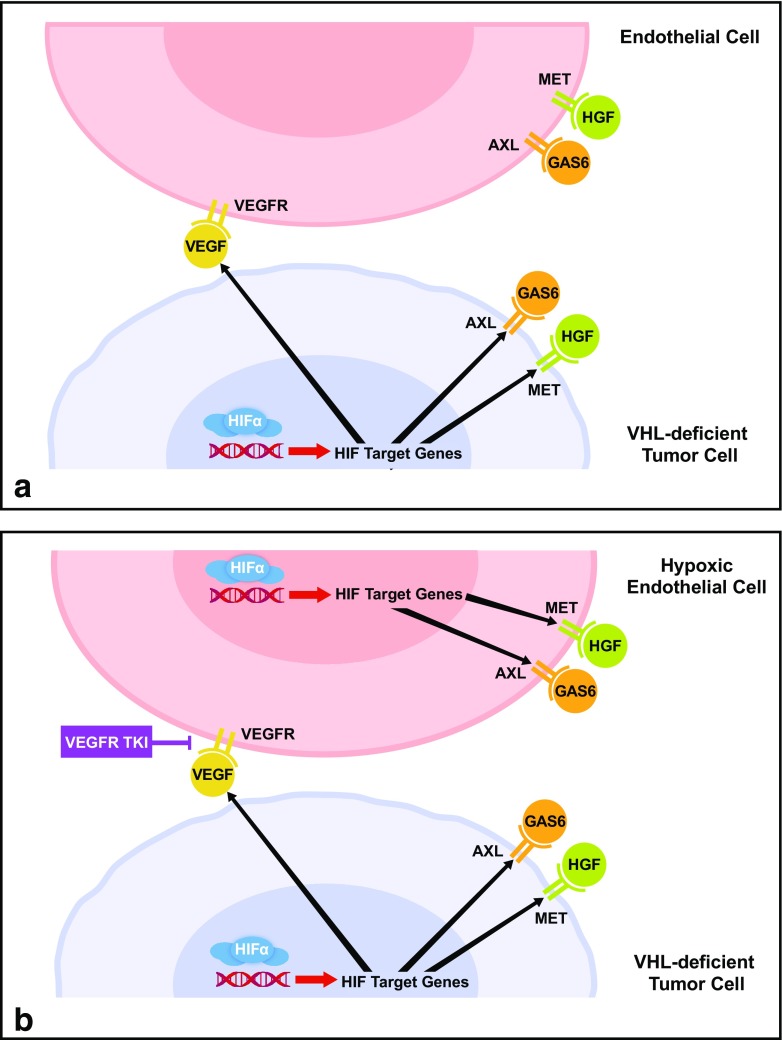
**a.** Activation of VEGFR, MET, and AXL signaling in RCC. VHL inactivation promotes stabilization of HIFα transcription factors and induces expression of hypoxic response genes, including VEGF, MET, and AXL. VEGF acts on endothelial cells to promote angiogenesis, and activation of MET and AXL signaling supports tumor growth, survival, invasion, and metastasis. **b.** Role of MET and AXL in resistance to VEGFR inhibitors. Resistance to VEGFR inhibitors results from upregulation of alternative angiogenic pathways, including MET and AXL. Increased expression of MET and AXL is due in part to induction of hypoxic response genes resulting from reduced angiogenesis. MET, AXL, and VEGF expression in tumor cells is also further upregulated in response to VEGFR inhibition. *HGF* hepatocyte growth factor, *HIF* hypoxia-inducible factor, *RCC* renal cell carcinoma, *TKI* tyrosine kinase inhibitor, *VEGFR* VEGF receptor

The *VHL* tumor suppressor is inactivated by mutation or epigenetic silencing in ~80% of sporadic clear cell RCC cases [29]. VHL deficiency in RCC drives angiogenesis, local invasion, and metastasis by activating the hypoxia-inducible factor (HIF)-regulated hypoxic response [17, 29]. At low oxygen concentrations, the transcription factors HIF1α/HIF2α induce expression of hypoxic response genes including angiogenic factors such as VEGF and PDGF, pro-invasive proteins that promote the epithelial-mesenchymal transition, and enzymes that support anaerobic metabolism. Under normoxia, VHL targets HIF1α/HIF2α for ubiquitin-dependent degradation by the proteasome. However, loss of VHL function leads to stabilization of HIF transcription factors and constitutive expression of their target genes (Fig. 1). In VHL-deficient tumor cells and endothelial cells, HIF2α rather than HIF1α is the main positive regulator of tumorigenesis and angiogenesis [29]. Activation of VEGFR signaling as a result of VHL deficiency underlies the clinical activity of anti-angiogenic agents in RCC.

VHL inactivation in RCC also induces overexpression and activation of the receptor tyrosine kinases MET and AXL [30–33]. MET protein expression is higher in all RCC subtypes compared with adjacent normal tissue, and higher MET expression correlates with shorter survival time in clear cell RCC [34]. Likewise, AXL mRNA is overexpressed in RCC tumors, and low AXL mRNA levels correlate with longer survival [35].

Aberrant MET activation promotes tumor growth, anti-apoptosis, angiogenesis, invasion, and metastasis [36, 37]. At the cellular level, MET and its cognate ligand hepatocyte growth factor (HGF) promote proliferation, survival, motility, and invasion. MET is essential for embryogenesis and tissue regeneration. During development, MET regulates tissue morphogenesis by inducing the epithelial-mesenchymal transition, a complex cellular reprogramming process which results in loss of cell-cell contacts, enhanced motility, and invasion. Activation of HGF/MET signaling in the endothelium induces branching morphogenesis and angiogenesis. In the malignant setting, dysregulation of these physiological processes drives both tumorigenesis and late-stage tumor progression.

AXL signaling is also implicated in tumor growth and survival [38, 39]. Activation of AXL by its cognate ligand GAS6 promotes cell proliferation, migration, and protection from apoptosis; in many contexts, AXL functions in concert with other receptors to amplify downstream signaling pathways.

Despite the success of anti-angiogenic agents in treating RCC, a fraction of patients do not respond to systemic therapy, and responding patients eventually progress and succumb to their disease. Resistance to VEGF-targeted therapy is mediated by upregulation of alternative angiogenic and invasive pathways, including MET and AXL [40–45] (Fig. 1). Chronic sunitinib treatment of RCC cell lines activates MET and AXL signaling, induces the epithelial-mesenchymal transition, and enhances cell migration, invasion, and angiogenesis [42, 43]. Likewise, sunitinib-resistant xenograft tumors display activated MET and AXL signaling and increased growth rates relative to sunitinib-sensitive controls [42, 44]. Mechanistically, sunitinib resistance in RCC can be mediated by competing non-coding RNAs that upregulate MET and AXL expression. Moreover, sunitinib resistance can be disseminated to sensitive cells by exosomes that transmit these regulatory RNAs [44].

## Preclinical Rationale and Early Clinical Trials

Recently published preclinical studies provide support for targeting VEGFR, MET, and AXL for RCC treatment in both first- and second-line settings. VHL-deficient tumor cell lines and patient-derived xenografts provide models for first-line therapy. In VHL-negative xenograft models, treatment with the VEGFR-selective inhibitor axitinib reduces tumor growth and prolongs survival, whereas treatment with crizotinib, which inhibits MET but not VEGFR, is much less effective. However, the combination of axitinib and crizotinib is significantly more effective than either treatment alone [45]. As described earlier, induced sunitinib resistance serves as a model for second-line therapy following anti-angiogenic agents. Targeting VEGFR, MET, and AXL is effective in this setting, as cabozantinib treatment of sunitinib-resistant xenograft tumors causes growth inhibition and regression [42]. Likewise, co-treatment with MET inhibitors or MET/AXL dual inhibitors overcomes resistance to VEGFR TKIs in xenograft models [44, 45].

Cabozantinib was first evaluated in patients with RCC in a phase 1b trial that also examined a potential drug-drug interaction with the CYP2C8 substrate rosiglitazone [46]. The starting dose of cabozantinib was 140 mg orally once per day. Among 25 RCC patients enrolled, 88% had received at least one prior therapy targeting the VEGF pathway. Cabozantinib demonstrated preliminary antitumor activity in the RCC cohort with an ORR of 28% (all partial responses), median PFS of 12.9 months, and median overall survival of 15.0 months. Dose reductions were employed in 80% of patients resulting in a median average daily dose of 75.5 mg, and the most common final daily dose was 60 mg.

## METEOR—Phase 3 Pivotal Study of Cabozantinib in RCC

Given the evidence from preclinical and early clinical studies, a randomized phase 3 trial (METEOR) was conducted to compare the efficacy and safety of cabozantinib with standard-of-care everolimus in patients with advanced RCC whose disease had progressed during or after prior VEGFR TKI therapy [21••, 22••]. Eligibility requirements included age ≥18 years, RCC with a clear cell histology component, measurable disease per Response Evaluation Criteria in Solid Tumors (RECIST) version 1.1 [47], and Karnofsky performance status ≥70%. Patients who had received previous mTOR inhibitor therapy were ineligible.

METEOR employed a trial-within-a trial design to provide adequate power for assessment of both the primary endpoint of PFS and the secondary endpoint of overall survival. The study was designed to enroll 650 patients, with a planned primary analysis of PFS in the first 375 randomized patients (the primary PFS population) after 259 events and a secondary analysis of overall survival in all 650 patients after 408 events. A total of 658 patients (the intent-to-treat [ITT] population) were randomized to receive open-label treatment with cabozantinib (*N* = 330) or everolimus (*N* = 328). Randomization was stratified by number of prior VEGFR TKIs and Memorial Sloan Kettering Cancer Center (MSKCC) risk groups [48]. Cabozantinib was initiated at 60 mg once daily, and everolimus was initiated at 10 mg once daily. The protocol specified dose interruptions or reductions as needed to manage treatment-emergent adverse events (AEs). Crossover between treatment arms was not allowed.

Baseline characteristics were balanced with the majority of patients (71%) having received only one prior VEGFR TKI. The most common prior therapies were sunitinib (63%) and pazopanib (43%). Notably, 31 patients (5%) had received prior nivolumab treatment. Bone metastases were reported in 22% of patients at baseline and visceral metastases in 74%. Most patients were categorized as having favorable (46%) or intermediate risk (42%) by MSKCC prognostic criteria.

At a data cut-off point of May 22, 2015 for the primary analysis, the minimum follow-up time was 11 months for the primary PFS population (*N* = 187 for cabozantinib and *N* = 188 for everolimus). The trial met its primary endpoint—cabozantinib treatment significantly improved PFS compared with everolimus with a median PFS of 7.4 versus 3.8 months (hazard ratio (HR) = 0.58, 95% confidence interval (CI) 0.45–0.75; *p* < 0.001). [21••] The PFS results were consistent in the ITT population with a median of 7.4 versus 3.9 months (HR = 0.51; 95% CI 0.41–0.62; *p* < 0.0001). Assessment of response in the ITT population by an independent radiology review committee showed an ORR of 17% for cabozantinib versus 3% for everolimus (*p* < 0.0001), all partial responses per RECIST v1.1. Stable disease was reported as the best overall response in 65% of cabozantinib-treated patients and 62% of everolimus-treated patients. Progressive disease was the best overall response in 12% of cabozantinib-treated patients and 27% of everolimus-treated patients. ORR as determined by investigators was 24% for cabozantinib and 4% for everolimus. At a second data cut-off of December 31, 2015, an information fraction of 78% was available for analysis of overall survival with a minimum follow-up of 13 months. Cabozantinib demonstrated a 34% reduction in the rate of death compared with everolimus, with a median overall survival of 21.4 versus 16.5 months (HR = 0.66, 95% CI 0.53–0.83; *p* = 0.00026). Efficacy outcomes for the ITT population in the METEOR trial are summarized in Table 1.

**Table 1:** Efficacy outcomes in intent-to-treat population of METEOR trial [21••, 22••]

Outcome	Cabozantinib (*N* = 330)	Everolimus (*N* = 328)	Hazard ratio (95% CI)	*p* value
Overall survival,^a^ months				
Median	21.4	16.5	0.66 (0.53–0.83)	0.00026
95% CI	18.7–NE	14.7–18.8		
Progression-free survival per IRC,^b^ months				
Median	7.4	3.9	0.51 (0.41–0.62)	<0.0001
95% CI	6.6–9.1	3.7–5.1		
Objective response per IRC^b,c^				
ORR, % (95% CI)^d^	17 (13–22)	3 (2–6)		<0.0001
Stable disease, %	65	62		
Progressive disease, %	12	27		
Objective response per investigator^b,c^				
ORR, % (95% CI)^d^	24 (19–29)	4 (2–7)		<0.0001
Stable disease, %	63	63		
Progressive disease, %	9	27		

*CI* confidence interval, *IRC* independent radiology review committee, *NE* not estimable, *ORR* objective response rate

^a^December 31, 2015 cutoff date

^b^May 22, 2015 cutoff date

^c^The sum of responses is less than 100% because there were patients with not evaluable or missing assessments in both arms

^d^Responses were all confirmed partial responses

Bone metastases are associated with poor outcomes in a number of solid tumors including RCC [49, 50]. MET, one of the targets of cabozantinib, is thought to play a role in modulating the activity of osteoblasts and osteoclasts, and preclinical data show that cabozantinib is active in bone metastasis models [24, 51, 52]. Subgroup analyses of the METEOR study population showed marked improvement for patients with bone metastases treated with cabozantinib versus everolimus for both PFS (HR = 0.33; 95% CI 0.21–0.51) and overall survival (HR = 0.54; 95% CI 0.34–0.84) [22••]. Furthermore, the rate of skeletal-related events was 16% for the cabozantinib arm versus 34% for the everolimus arm among those with a history of skeletal-related events at baseline [53].

Given the number of available treatments for RCC, optimizing the sequence of targeted therapies has become a relevant issue. In the METEOR study, the benefit of cabozantinib was maintained regardless of the prior therapy received by the patient. Cabozantinib demonstrated improvements over everolimus for PFS and overall survival across multiple subgroups, including those defined by number of prior VEGFR TKIs (1 or ≥2), by duration of treatment with first VEGFR TKI (≤6 or >6 months), by prior treatment with sunitinib or pazopanib as the only VEGFR TKI, and by prior treatment with a PD-1/PD-L1 checkpoint inhibitor [22••, 54].

Safety analyses of all-causality treatment-emergent AEs showed that 21% of patients receiving cabozantinib experienced a grade 1/2 AE versus 32% for those receiving everolimus at the December cut-off, the grade 3 AE rate was 63 versus 52%, and the grade 4 AE rate was 8% in both treatment arms [22••]. The most common grade 3 AEs experienced by patients in the cabozantinib group were hypertension (15 vs 4% in the everolimus group), diarrhea (13 vs 2%), fatigue (11 vs 7%), and palmar-plantar erythrodysesthesia syndrome (8 vs 1%). The most common grade 4 event in the cabozantinib arm was hypomagnesemia (3% versus none in the everolimus arm), with all other grade 4 events <1%. Dose reductions were utilized to manage AEs in 62% of patients in the cabozantinib arm and 25% of patients in the everolimus arm. Treatment-related deaths were rare in both arms—one death in the cabozantinib arm (not otherwise specified by the investigator) and two in the everolimus arm (one due to aspergillus infection and one due to aspiration pneumonia).

In brief, cabozantinib treatment significantly improved all three key efficacy endpoints of overall survival, PFS, and ORR compared with everolimus in the METEOR trial with an acceptable safety profile. Furthermore, the improvements in overall survival and PFS associated with cabozantinib remained consistent across subgroups of patients, including established prognostic risk groups (i.e., MSKCC) [22••] as well as other pre-specified patient subgroups such as those defined by bone metastases or prior therapy [53, 54].

## Current Landscape and Future Directions

Within the last year, the immune checkpoint inhibitor nivolumab and the combination of lenvatinib plus everolimus also received FDA approval as second-line RCC treatments after anti-angiogenic therapy. In a phase 3 randomized trial comparing nivolumab with everolimus, nivolumab treatment improved overall survival (HR = 0.73; 95% CI 0.60–0.89; *p* = 0.0018) and improved ORR, but did not improve PFS [18••, 55]. In a phase 2 randomized trial, lenvatinib plus everolimus demonstrated improved PFS (HR = 0.37; 95% CI 0.22–0.62, per investigators) and improved ORR relative to everolimus monotherapy [56]. For overall survival, the HR for lenvatinib plus everolimus compared with everolimus was 0.67 (95% CI 0.42–1.08) [56].

The recent FDA approvals of nivolumab, cabozantinib, and lenvatinib plus everolimus have substantially expanded the available treatment options for patients with previously-treated RCC, and have altered the therapeutic landscape. Given these developments, the National Comprehensive Cancer Network (NCCN) Guidelines for Kidney Cancer (Version 3.2016) provided the following category 1 recommendations which are based on “high-level evidence where there is uniform NCCN consensus that the intervention is appropriate” [11]: For advanced RCC with predominant clear-cell histology, category 1 recommendations for first-line therapy are sunitinib, bevacizumab plus interferon-α, pazopanib, and temsirolimus (for poor prognosis patients). For subsequent treatment after anti-angiogenic therapy, category 1 recommendations are axitinib, cabozantinib, nivolumab, and everolimus. Cabozantinib and nivolumab are preferred over everolimus based on the results of phase 3 trials which demonstrated improved overall survival.

While these advances have increased treatment options for patients, they also raise questions such as how to treat specific patient populations and how to sequence therapies. The availability of drugs with different mechanisms of action (VEGF-targeted agents, spectrum-selective TKIs that target VEGFRs, mTOR inhibitors, and immune checkpoint inhibitors) provides multiple options for second-line and later-line therapies. To further expand available therapies, approved second-line agents are being evaluated in the first-line setting, and regimens combining agents with different molecular mechanisms are being explored.

Cabozantinib is currently being evaluated for RCC treatment in three additional clinical settings: first-line therapy versus sunitinib, combination therapy with immune checkpoint inhibitors, and treatment of papillary RCC. In the randomized phase 2 CABOSUN trial (NCT01835158), cabozantinib demonstrated a statistically significant improvement in PFS and ORR versus sunitinib in previously untreated intermediate or poor risk patients with advanced clear cell RCC [57]. In a phase 1b trial, the combination of cabozantinib and nivolumab with or without ipilimumab is being assessed in patients with metastatic genitourinary tumors, including RCC (NCT02496208) [58]. The primary objectives of this trial are evaluation of the tolerability of the combinations, identification of any dose-limiting toxicities, and determination of the recommended phase 2 doses for the combinations. Secondary objectives include assessing the effect of the combinations on ORR, PFS, and overall survival. MET is also a potential target in non-clear-cell RCC because it is mutated in type I papillary RCC and overexpressed in both papillary subtypes [59, 60]. Based on this biological rationale, a randomized phase 2 trial was recently initiated in metastatic papillary RCC to compare the efficacy of TKIs that target VEGFRs, MET, or VEGFRs plus MET (sunitinib, crizotinib, savolitinib, and cabozantinib) (NCT02761057) [61]. PFS is the primary endpoint of the trial, and ORR, overall survival, and safety are the secondary outcomes.

## Conclusion

Cabozantinib is a new standard of care for patients with advanced RCC following anti-angiogenic therapy. In the METEOR trial, cabozantinib demonstrated statistically significant improvements compared with everolimus in the three key efficacy endpoints of overall survival, PFS, and ORR. Treatment benefit was maintained across multiple patient subgroups including risk category, nature of prior therapy, and extent of tumor burden. Ongoing clinical trials have the potential to expand the clinical utility of cabozantinib in advanced RCC to first-line therapy or treatment of non-clear-cell RCC.
